# *TDT-HET*: A new transmission disequilibrium test that incorporates locus heterogeneity into the analysis of family-based association data

**DOI:** 10.1186/1471-2105-13-13

**Published:** 2012-01-20

**Authors:** Douglas Londono, Steven Buyske, Stephen J Finch, Swarkar Sharma, Carol A Wise, Derek Gordon

**Affiliations:** 1 Department of Genetics and Human Genetics Institute, Rutgers, The State University of New Jersey, 145 Bevier Road, Piscataway, NJ, 08854 USA; 2 Department of Statistics & Biostatistics, Hill Center, Rutgers, The State University of New Jersey, 110 Frelinghuysen Road Piscataway, NJ 08854-8019 USA; 3 Department of Applied Mathematics and Statistics, Stony Brook University, Stony Brook, NY, 11794-3600 USA; 4 Texas Scottish Rite Hospital for Children, 2222 Welborn Street, Dallas, TX 72519 USA; 5 Department of Orthopedic Surgery and McDermott Center for Human Growth and Development, University of Texas Southwestern Medical Center, 5323 Harry Hines Boulevard, Dallas, TX 75390 USA

## Abstract

**Background:**

Locus heterogeneity is one of the most documented phenomena in genetics. To date, relatively little work had been done on the development of methods to address locus heterogeneity in genetic association analysis. Motivated by Zhou and Pan's work, we present a mixture model of linked and unlinked trios and develop a statistical method to estimate the probability that a heterozygous parent transmits the disease allele at a di-allelic locus, and the probability that any trio is in the linked group. The purpose here is the development of a test that extends the classic transmission disequilibrium test (*TDT*) to one that accounts for locus heterogeneity.

**Results:**

Our simulations suggest that, for sufficiently large sample size (1000 trios) our method has good power to detect association even the proportion of unlinked trios is high (75%). While the median difference (*TDT-HET *empirical power - *TDT *empirical power) is approximately 0 for all MOI, there are parameter settings for which the power difference can be substantial. Our multi-locus simulations suggest that our method has good power to detect association as long as the markers are reasonably well-correlated and the genotype relative risk are larger. Results of both single-locus and multi-locus simulations suggest our method maintains the correct type I error rate.

Finally, the *TDT-HET *statistic shows highly significant p-values for most of the idiopathic scoliosis candidate loci, and for some loci, the estimated proportion of unlinked trios approaches or exceeds 50%, suggesting the presence of locus heterogeneity.

**Conclusions:**

We have developed an extension of the *TDT *statistic (*TDT-HET*) that allows for locus heterogeneity among coded trios. Benefits of our method include: estimates of parameters in the presence of heterogeneity, and reasonable power even when the proportion of linked trios is small. Also, we have extended multi-locus methods to *TDT-HET *and have demonstrated that the empirical power may be high to detect linkage. Last, given that we obtain PPBs, we conjecture that the *TDT-HET *may be a useful method for correctly identifying linked trios. We anticipate that researchers will find this property increasingly useful as they apply next-generation sequencing data in family based studies.

## Background

In genetics, heterogeneity is a major feature of human traits. Genetic heterogeneity occurs when the same or clinically indistinguishable phenotypes are caused by different genetic factors. This can be due to multiple variants located in the same locus (allelic heterogeneity) or to mutations located in different loci (locus heterogeneity).

The focus of this work is locus heterogeneity, specifically heterogeneity caused by having an unknown subset of pedigrees in a sample being unlinked to a disease locus while the rest are linked [1,2].

There are many reported examples of locus heterogeneity, including breast cancer [3-6], maturity-onset diabetes of the young (MODY) [7], epilepsy [8], early-onset Alzheimer's Disease [9], rheumatoid arthritis [10], non-polyposis colorectal cancer [11], non-syndromic hearing loss [12-14] and retinitis pigmentosa [15-17].

Locus heterogeneity can substantially affect the power of linkage and association analyses [18-27]. In linkage analysis, there are many examples of methods that address this issue. For example, we have: the M test [28] (also known as K-test [29,30]), a likelihood ratio test (LRT) that estimates the value of the (assumed fixed) recombination fraction *(θ) *for each pedigree in a sample; the B-test [29], which is a more powerful version of the M-test that assumes an underlying beta null distribution for each estimated *θ*; the admixture test (A-test), which is based on the difference between the log-likelihood of the admixture model (data are composed of linked and unlinked families) and the homogeneity model (families are all linked with a common *θ*) [2,31-36]; the D-test [30], a combination of the A and B tests and finally, the C-test [19], which is based on the M-test and for which the underlying null probability distribution is determined by simulation. The M and B tests were originally developed to identify different values of *θ *for different pedigrees. For the A-test, families are grouped into two types: a proportion *a *that are linked to the disease locus (*θ *< 1/2) and a proportion 1- *α *that are unlinked (*θ *= 1/2) [1,2]. As contrasted with M and the B tests, which place pedigrees into classes a priori, the A test accounts for heterogeneity by maximizing the standard log-odds (LOD) score [37] over *α *and *θ*. That is, each pedigree has some probability of being in the linked or unlinked group. This statistic is known as the heterogeneity LOD score (HLOD) [38].

The A-test has been implemented in a suite of programs to test for heterogeneity vs. homogeneity (HOMOG) [38]. More complex heterogeneity scenarios are also available in this package: HOMOG1 allows for gender specific differences in *θ*. HOMOG2, HOMOG3, HOMOG4, distinguish two, three and four types of families respectively, each linked to different disease loci on the same chromosome. HOMOG3R is a special case of HOMOG3 where there are three family classes: the first class is linked to a given marker; the second is linked to another marker on a different chromosome and the third is linked to neither marker. Lastly, HOMOGM [39], an extension of HOMOG3R, allows for any number of disease loci.

It is important to mention linkage analysis methods for quantitative trait loci (QTL) that account for locus heterogeneity in the analysis. Yang et al. [40] proposed a QTL mapping model for sib pair data. Knight et al. [41] and Ekstrøm et al. [42] independently developed LRT-based models in which the underlying null probability distributions are determined by simulation while Wang and Peng [43] proposed three test statistics with known null asymptotic distributions. It appears that relatively fewer publications considering locus heterogeneity for association have been published as compared with heterogeneity for linkage. When using the search terms "(locus heterogeneity) AND (linkage)" in ISI Web of Knowledge, we retrieve a total of 2,418 titles. By contrast, using the using the search terms "(locus heterogeneity) AND (association)", we retrieve a total of 884 titles, an almost 67% reduction. Having documented that, we do note that methods to address locus heterogeneity for association-based methods have been developed.

Latent class models [44] have been used to estimate membership-class probabilities for individuals with similar genetic backgrounds [45-48].Ordered Subset Analysis (OSA)-based models have been extended to association, including the sequential addition (SA) procedure [49] and the OSA case-control (OSACC) method [50]. For family-based data, the OSA-*TDT *[51] applies OSA to the transmission disequilibrium test (*TDT*) [52], and the APL-OSA [53] similarly applies OSA to the "association in the presence of linkage" test (APL) [54].

Yang et al. [55] extended the Posterior Probability of Linkage (PPL) method to one that incorporates linkage disequilibrium information between marker and disease alleles. Huang et al. [56] extended the PPL method to case-control data. These methods maintain all the features of the original PPL method for linkage, namely, they do not require correction for multiple testing and they can sequentially update information across multiple data sets.

Wang and Huang [22] developed two LRT extensions of the HLOD: the LD-Het for general pedigrees and the LD-multinomial for affected sib pair data. Here, LD stands for linkage disequilibrium. Schmidt et al. [57] proposed using a two-stage linkage/association approach for affected sib pair data. Finally, Zhou and Pan [58] used a mixture model to allow for locus heterogeneity in a case-control design.

The purpose of this work is the development of a new test statistic that we call *TDT-HET*, that allows for locus heterogeneity when applying the *TDT *statistic. This work is largely motivated by the recent work of Zhou and Pan [58]. As in their paper, our statistic is based on an underlying mixture model. We apply an expectation-maximization (EM) algorithm to compute log-likelihoods of the data under null and alternative hypotheses. The EM algorithm also produces maximum likelihood estimates of parameters such as the probability that a heterozygous parent transmits the disease allele to an affected child, the probability that a trio (mother, father, affected child) is linked to the locus in question, and the probability that certain trio types (determined by the constellation of genotypes) are linked to the locus being studied. In addition, we extend our *TDT-HET *method to statistic that can evaluate multiple loci jointly. This extension is motivated by and similar to the work of Hoh, Ott, and colleagues. They called their method *SumStat *[59-62].

For both single-locus and multi-locus simulations, we evaluate the type I error rate and the power of the *TDT-HET *method to detect association. In addition, we apply the *TDT-HET *method to candidate loci from a study of idiopathic scoliosis trios to determine if there is any suggestion of locus heterogeneity at the loci considered, and whether the results suggest evidence for association in the presence of heterogeneity.

## Methods

### Notation

Much of the notation we use comes from the work of Zhou and Pan [58], who developed a test statistic for case-control data that allows for locus heterogeneity. Also, much of the *TDT *notation comes from the work of Schaid and Sommer [63]. Here we present notation used in the main body of this work. A fuller notation list may be found in the additional file 1, Appendix (Notation section).

M = The disease allele at the putative disease SNP locus.

N = The non-disease allele at the putative disease SNP locus.

*x*_*abc *_= The trio where parent 1, parent 2, and affected child have *a*, *b*, and *c *copies of the M allele at the putative disease locus (range for all copies: 0 - 2). For example, *x*_222 _is the trio with mating type MM × MM and affected child genotype MM. Throughout this work, we will use the notation *abc *interchangeably with *x*_*abc*_.

*n*_*abc *_= The number of trios *x*_*abc *_in the sample.

*n *= The total number of trios in the study.

*D *= Event that the child in a trio is affected.

*A *= Event that individual in a population is affected.

*ϕ *= Pr(*A*) = Disease prevalence.

*f*_*i *_= Pr(*A*|*i *copies of M allele in individual's genotype) = Disease penetrances, *i *= 0,1,2.

$R_{i}=\frac{f_{i}}{f_{0}}$, *i *= 1,2 = genotype relative risks (GRR) [63]. *R*_1 _corresponds to the heterozygote GRR and *R*_2 _to the GRR for disease allele homozygote. We consider three kinds of disease modes of inheritance (MOI) in this work: *R*_1 _= *R*_2 _(dominant); *R*_1 _= 1 (recessive); $R_{1}^{2}=R_{2}$ (multiplicative).

*p *= Pr(*M*) = Disease allele frequency (DAF).

*q *= Pr(N) = 1 - *p *= Non-disease allele frequency.

*t *= Pr(heterozygous parent transmits M allele to affected offspring). In this work, the null hypothesis, *H*_0_, is *t *= 0.5. The alternative hypothesis, *H*_1_, is *t *≠ 0.5.

*μ*_*k*,i _= Pr(Mating type = *i*|*D*, pop = *k*) = probability that the mating type is *i *given that the child is affected and the trio comes from the *k*^th ^population, 1 ≤ *k *≤ 2. Throughout this work, we shall use the notation *k *= 1 to indicate that the trio is in the linked population (*t *≠ 0.5) and *k *= 2 to indicate that the trio is in the unlinked population (*t *= 0.5). Similar to Schaid and Sommer [63], we consider 6 mating types in this work. We recognize that other models, such as those considered by Weinberg and colleagues [64,65], require more than six mating type frequencies. We conjecture that our work extends to such situations.

*π*_1 _= Pr(trio is linked to trait locus) = Pr(*t *≠ 0.5). In this work we specify that *t *is the same for all linked trios. This specification is also made for the recombination fraction in some tests of linkage allowing for heterogeneity (see, e.g., work by C. A. B. Smith [1,2] and Ott [38], specifically the method implemented in programs such as HOMOG [38], GENEHUNTER [66], SIMWALK2 [67], VITESSE [68], MERLIN [69], and other programs).

$$\pi_{2}=1-\pi_{1}.$$

$\hat{x}$ = Maximum likelihood estimate (MLE) of the parameter *x*. This MLE is determined by means of the EM algorithm.

*z*_*k,j *_= The indicator variable for population *k *and trio *x*_*j*_, where the subscript *j *indicates the *j*^th ^trio in the sample.

$\tau_{k,j}^{\left(r\right)}=r^{\text{th}}$ iteration step estimate that the *j*^th ^trio is in the *k*^th ^population, *k *= 1,2. Without loss of clarity, we will use sometimes write $\tau_{k,abc}^{\left(r\right)}$, where *abc *refers to the trio *x*_*abc *_(see above).

### *TDT-HET *Test Statistic

The TDT-HET statistic is a likelihood ratio statistic. Log-likelihoods under the null hypothesis, H_0_: *t *= 0.5 or *π*_1 _= 0, and under the alternative hypothesis,H_1 _: *t *≠ 0.5 and *π*_1 _≠ 0, are computed by maximizing these parameters for the observed data. We compute the maximum likelihood estimates under H_0 _and H_1 _using the Expectation-Maximization method [70]. P-values are computed using permutation methods. Full details are provided in the additional file 1, Appendix (TDT-HET Statistic section).

*All trios drawn from a population with one set of parental mating types*

### Simulations

#### Single locus

To evaluate the type I error rate and power of the test statistic under different scenarios, we perform simulations. In this section, we describe simulations where we consider type I error rate and power for a single disease locus that has been genotyped. The parameter settings that we consider are presented in Table 1.

**Table 1:** Simulation parameter settings for the single-locus simulations

Item	Parameter	Setting
**1**	MOI	Dominant, Recessive, Multiplicative
***2***	*ϕ*	0.05, 0.15
**3**	*R*_*2*_	1.0 (Null), 2.25
**4**	*π*_*1*_	0.25, 0.50, 0.75, 1.0
**5**	*p*	0.10, 0.25, 0.50, 0.75, 0.90
**6**	Number of trios	1000
**7**	Number of permutations per statistic	500
**8**	Number of starting points	200
**9**	Number of EM steps per starting point	100
**10**	*ε*	10^-6^
**11**	Penalty *C *in EM algorithm (Equation (1))	0.001
**12**	Number of replicates per vector (Items 1-3)	250

MOI = Mode of inheritance

*ϕ *= Disease prevalence

*R*_*2 *_= Genotype relative risk for disease allele homozygote

*π*_*1 *_= Proportion of linked trios

*p *= Disease allele frequency

*ε *= Tolerance

We comment that, in item 3 in Table 1, we specify that the disease locus is in Hardy Weinberg Equilibrium. In our simulations, we use the value *p *to determine the mating type frequencies. Specifically, we specify random mating in the single-locus simulations, so that the mating-type frequencies *μ*_*i *_are the products of the parental genotype frequencies, which themselves are determined by the allele frequency *p *according to Hardy-Weinberg Equilibrium. For example, the frequency of the mating-type MN × NN is 2 × (2*pq*) × *q*^2 ^= 4*pq*^3^, where *q *= 1 - *p *is the frequency of the N allele. Schaid and Sommer provide similar results in their Table 1[63]. While we do not simulate non-Hardy-Weinberg situations in our single-locus simulations, we do so in our multi-locus simulations (see below).

#### Multi-locus

To evaluate the *TDT-HET *statistic for multiple loci, we apply a slight variant of the *"SumStat *" procedure developed by Hoh, Ott, and colleagues [59-62]. While these researchers consider sums of ever-increasing number of SNPs, in this work, we consider just the full sum. Specifically, for each of the *k *loci, 1 ≤ *k *≤ *L*, where *L *is the number of loci in the simulation, we compute *TDT-HET*(*k*), the value of the statistic at the *k*^th ^locus. We then compute:

(1) $$SumStat=\sum_{k=1}^{L}TDT-HET\left(k\right).$$

Empirical significance levels are determined through permutation. Since each locus *k *has 500 permuted *TDT-HET*(*k*) statistics associated with it, we can compute the permuted *SumStat *statistics for each permutation number (1 to 500) (This value changes to 100,000 for the Idiopathic scoliosis Candidate Loci - see below). The empirical significance level is defined as the proportion of *SumStat *values that exceed the *SumStat *value for the observed data. Table 2 lists the parameter settings for our multi-locus simulations (those parameters not listed there are the same as listed in Table 1).

**Table 2:** Simulation parameter settings for the multi-locus simulations

Item	Parameter	Setting
**1**	Number of loci	4
**2a**	Locus transmission probability: MOI	Multiplicative
**2b**	Locus transmission probability: *p*	0.10, 0.50, 0.9
**3**	*R*_*2*_	1.0 (Null), 2.25,9.0
**4**	*π*_*1*_	0.25, 0.75
**5**	*ρ*	0.8

MOI = Mode of inheritance

*p *= Disease allele frequency

*R*_*2 *_= Genotype relative risk for disease allele homozygote

*π*_*1 *_= Proportion of linked trios

*ρ *= correlation coefficient. *ρ *= 1 (perfect correlation), *ρ *= 0 (no correlation)

Here, we consider 4 correlated SNPs in each simulation. Mating types for the first locus (labeled *MT*[1] [*i*]) are determined using the disease allele frequency *p *(setting 2b in Table 2). As above, each mating type *i *= 1, ..., 6 will have its frequency determined using HWE proportions. See above (Simulations - Single Locus) for formulas determining mating type frequencies for the first locus. For each consecutive locus *l*, 2 ≤ *l *≤ 4, the mating type frequencies for the *i*^th ^mating type is determined in the following fashion.

1. Define [*l*][*i*] = *ρ *× *MT*[*l *- 1][*i*] + (1 - *ρ*) × *X*, where *X*~*U*(0,1).

2. Compute $SumY\left[l\right]=\sum_{i=1}^{6}Y\left[l\right]\left[i\right]$.

3. $MT\left[l\right]\left[i\right]=\frac{Y\left[l\right]\left[i\right]}{SumY\left[l\right]}$.

Note that, if *ρ *= 1 (perfect correlation), then the mating type frequencies for each locus are identical. If *ρ *= 0 (no correlation), then each locus has mating type frequencies that are essentially random numbers that sum to 1. In the Results section, power is computed at the 1% significance level (see below).

### Idiopathic Scoliosis Candidate Loci

We applied our method to a dataset that included selected loci from our published genome-wide association study (GWAS) of adolescent idiopathic scoliosis (AIS) [71]. Briefly, AIS is a common spinal deformity with a prevalence of ~3% in school age children worldwide. The underlying genetics of AIS are generally complex and heterogeneity is apparent [71,72]. In the work presented here we selected genotypes for five loci derived in a total of 447 trios (1849 samples) from 447 families that were included in our previous publication [71]. Of the five loci, four (rs1400180, rs10510181, rs1040315, and rs2222973) were selected due to their significance by *TDT *analysis, their evidence of clustering, and their proximity to genes of potential biological relevance. We also selected an additional locus, rs11770843, because of its proximity to haplotypes previously linked and associated with AIS [73].

While we keep a number of the settings fixed (Table 1, settings 8-9), we alter the number of permutations per statistic to 100,000. Note that this number is much larger than the number performed in our simulation studies. The reason for this is that we are analyzing far fewer markers here than in our simulations, so time/CPU constraints are not really an issue. Also, the *SumStat *P-value is based on 100,000 permutations, since we have 100,000 permutation *TDT-HET *statistics for each locus.

As a comparison, we compute the *TDT *statistic [52] as implemented in the PLINK software [74]. We also compute point-wise and family-wise permutation p-values (labeled Emp1 and Max(T), respectively by Purcell et al. [74]). The Max(T) permutation statistic is based on the maximum observed test per permutation and so accurately reflects the family-wise error rate in the presence of LD.

While this description is for a genome-wide study, we consider only the situation max(T) applied to 5 candidate SNPs. We compare the max(T) statistic to our Bonferroni-corrected maximum *TDT-HET SumStat *statistic (corrected over 2 chromosomes, since one chromosome has one locus).

## Results

### Simulations

#### Null hypothesis (Type I error rate)

***R***_**2 **_**= 1**. **0**

**Figure 1:**
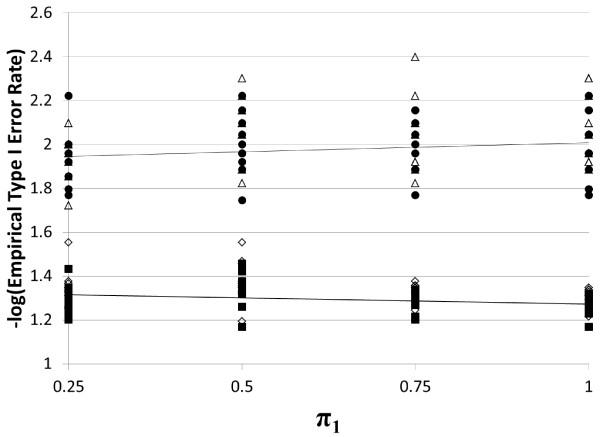
**Empirical type I error rates for *TDT-HET *and *TDT *statistics**. Here we present the empirical type I error rates for the *TDT-HET *and *TDT *statistics for different settings of the prevalence (0.05 or 0.15), DAF (0.10, 0.25, 0.50, 0.75, 0.90), *π*_1 _(0.25, 0.50, 0.75, 1.00) at two different significance levels (5%, 1%). The various plotted shapes in the figure represent empirical type I error rates (-log-transformed) for a fixed setting of the parameters. Solid square = *TDT-HET*, 5% Empirical Type I Error Rate. Hollow diamond = *TDT*, 5% Empirical Type I Error Rate. Solid circle = *TDT-HET*, 1% Empirical Type I Error Rate. Hollow triangle = *TDT*, 1% Empirical Type I Error Rate.

For the situation where *R*_2 _= 1.0, we present empirical type I error rates at the 5% and 1% levels in Figure 1. The minimum observed type I error rate at the 5% level for *TDT-HET *is 0.04 (-log(0.04) = 1.46), which occurs for the settings: *ϕ *= 0.05, *π*_1 _= 0.50, *p *= 0.10, and the maximum observed type I error rate is 0.07 (-log(0.07) = 1.17), which occurs for the settings: *ϕ *= 0.15, *π*_1 _= 0.50, *p *= 0.75. The median type I error rate is 0.05.

At the 1% level, the minimum observed type I error rate for *TDT-HET *is 0.006 (-log(0.006) = 2.22), which occurs for the settings: *ϕ *= 0.05, *π*_1 _= 0.25, *p *= 0.25, and the maximum observed type I error rate is 0.02 (-log(0.02) = 1.74), which occurs for the settings: *ϕ *= 0.15, *π*_1 _= 0.50, *p *= 0.75. The median type I error rate is 0.01.

Given that the type I error rate is computed over 250 replicates for each simulation vector setting in Table 1, we can use the method implemented in the BINOM program [38] to compute exact 95% confidence intervals for each empirical type I error rate. For the minimum and maximum empirical rates presented above from Figure 1, BINOM indicates that 0.05 and 0.01 are contained in in each respective 95% confidence interval. In addition, in Figure 1 we include linear trend lines using the method implemented in the MS Office 2007 Excel Spreadsheet software. Note that the 5% and 1% trend lines are very close to the constant lines y = 1.30 and y = 2.00, which are the -log-transformed values of 0.05 and 0.01, respectively. This result suggests that the *TDT-HET *maintains the correct type I error rate under the null hypotheses.

As a confirmation of our simulation code, we comment that the minimum observed type I error rate at the 5% level for *TDT is *0.03 (-log(0.03) = 1.55), the maximum observed type I error rate is 0.06 (-log(0.06) = 1.19), and the median type I error rate is 0.05. At the 1% level, the minimum observed type I error rate for *TDT is *0.004 (-log(0.004) = 2.40), the maximum observed type I error rate is 0.02 (-log(0.02) = 1.72), and the median type I error rate is 0.01. These results suggest that our simulation code is correctly simulating null data.

### Alternative hypotheses (Power)

#### Single Locus

**Figure 2:**
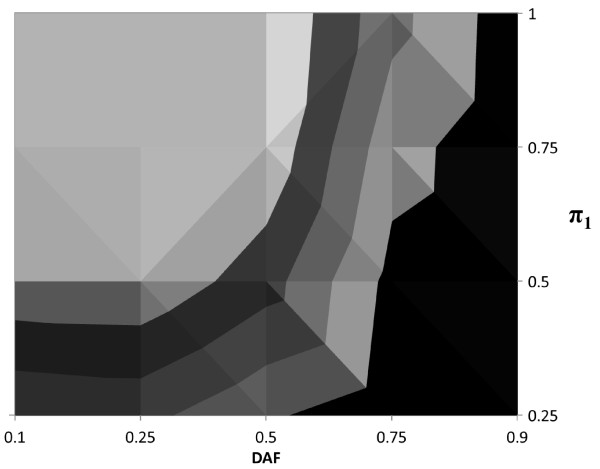
**Contour plot of *TDT-HET *empirical power for Dominant MOI at 5% significance level**. Empirical powers at the 5% significance level for prevalence (*ϕ*) equal to 0.05; DAF (*p*) equal to 0.10, 0.25, 0.50, 0.75, 0.90 and *π*_1 _equal to 0.25, 0.50, 0.75, 1.00. Each contour in the figure represents a range of empirical power values. There are five contours, corresponding to power ranges (*x*, *x *+ 0.20), where *x *= 0.00, 0.20, 0.40, 0.60, 0.80. For example, the black contour represents the power range (0.00, 0.20). The light gray contour contiguous to the black contour represents the power range (0.20, 0.40) and so forth. The lightest contour represents the power range (0.80, 1.00).

**Figure 3:**
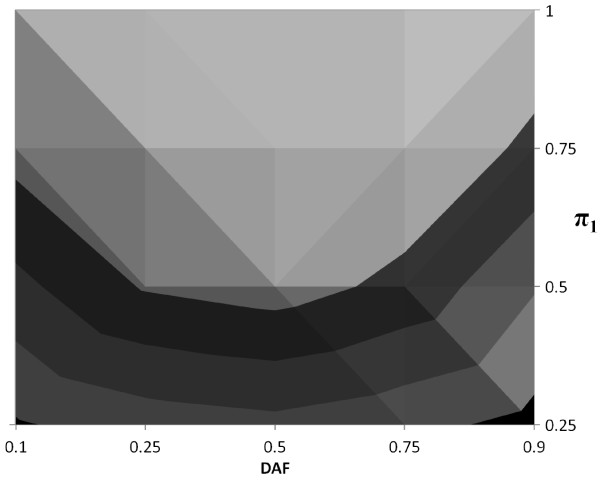
**Contour plot of *TDT-HET *empirical power for Multiplicative MOI at 5% significance level**. Empirical powers at the 5% significance level for prevalence (*ϕ*) equal to 0.05; DAF (*p*) equal to 0.10, 0.25, 0.50, 0.75, 0.90 and *π*_1 _equal to 0.25, 0.50, 0.75, 1.00. Each contour in the figure represents a range of empirical power values. There are five contours, corresponding to power ranges (*x*, *x *+ 0.20), where *x *= 0.00, 0.20, 0.40, 0.60, 0.80. For example, the black contour represents the power range (0.00, 0.20). The light gray contour contiguous to the black contour represents the power range (0.20, 0.40) and so forth. The lightest contour represents the power range (0.80, 1.00).

**Figure 4:**
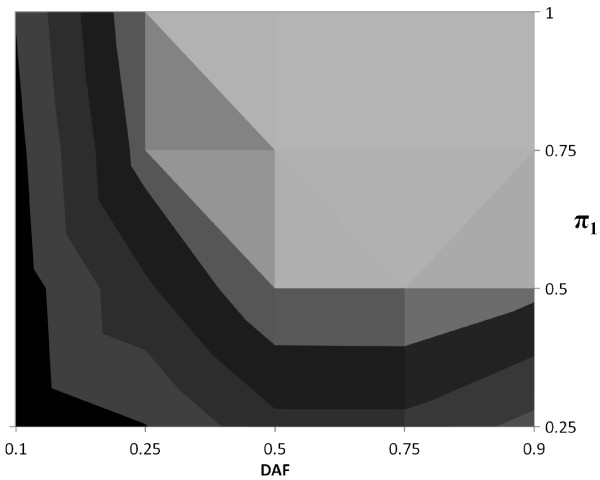
**Contour plot of *TDT-HET *empirical power for Recessive MOI at 5% significance level**. Empirical powers at the 5% significance level for prevalence (*ϕ*) equal to 0.05; DAF (*p*) equal to 0.10, 0.25, 0.50, 0.75, 0.90 and *π*_1 _equal to 0.25, 0.50, 0.75, 1.00. Each contour in the figure represents a range of empirical power values. There are five contours, corresponding to power ranges (*x*, *x *+ 0.20), where *x *= 0.00, 0.20, 0.40, 0.60, 0.80. For example, the black contour represents the power range (0.00, 0.20). The light gray contour contiguous to the black contour represents the power range (0.20, 0.40) and so forth. The lightest contour represents the power range (0.80, 1.00).

In Figures 2, 3 and 4 we present contour plots of empirical powers of the *TDT-HET *method for a single locus at the 5% significance level for dominant, multiplicative and recessive MOIs, respectively. The contour plots provide empirical power ranges as a function of the proportion of linked trios *π*_1 _and DAF (*p*), where settings of the input values are stated in Table 1, items 4 and 5). Other parameter settings that are used in these simulations are also provided in Table 1. In each of these figures, the prevalence is 0.05.

Each contour in each figure represents a range of empirical power values. In each figure, there are five contours, corresponding to power ranges (*x*, *x *+ 0.20), where *x *= 0.00, 0.20, 0.40, 0.60, 0.80. For example, the black contour represents the power range (0.00, 0.20). The light gray contour contiguous to the black contour represents the power range (0.20, 0.40) and so forth. The lightest contour represents the power range (0.80, 1.00).

Studying these figures, we can draw a number of conclusions. First, we see that, independent of the disease MOI, as the proportion of linked trios *π*_1 _increases, the empirical power increases as well. This result is not surprising. It is interesting to note that power for a fixed DAF is very much dependent upon disease MOI. For example, we see in Figure 2 that empirical power for a dominant MOI tends to be larger when *p *≤ 0.50. For a multiplicative MOI (Figure 3), empirical power tends to be larger for 0.25 ≤ *p *≤ 0.75. Finally, for a recessive MOI (Figure 4), MOI tends to be larger when *p *≥ 0.50.

**Figure 5:**
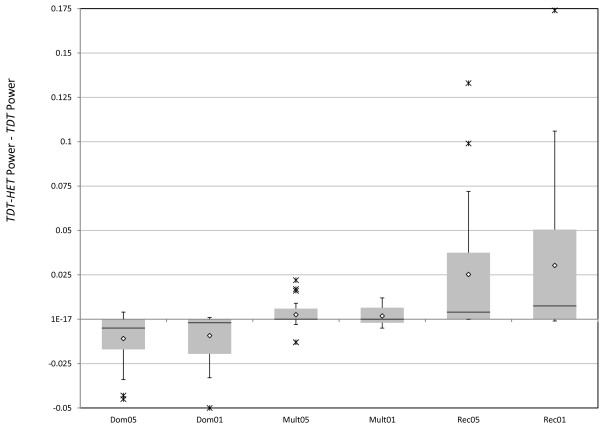
**Box and whiskers plots of differences (*TDT-HET *empirical power - *TDT *empirical power) for different disease MOIs and significance levels**. Here we present Box and Whisker plots for the differences (*TDT-HET *Empirical Power - *TDT *Empirical Power) for the three MOIs and the two significance levels. The code referring to each plot is: (MOI, Significance Level). For example, Dom05 refers to the differences for the dominant MOI at the 5% significance level. Similarly, Dom01 refers to the differences for the dominant MOI at the 1% significance level. For each gray box, the upper side indicates the 3rd quartile value, the lower side indicates the 1st quartile value, and the black horizontal line in the middle of the box indicates the median value. Mean values are indicated by white diamonds, and outliers are indicated by stars.

*How does the TDT-HET statistic's power compare with that of the TDT in the presence of heterogeneity? *Our previous work determining the non-centrality parameter of the *TDT *in the presence of heterogeneity [27] allows us to answer this question directly. However, we use *TDT *empirical power instead to compare "apples to apples" for power. In Figure 5, we present a Box and Whiskers plot [75] that reports a summary of the distribution of differences (*TDT-HET *empirical power - *TDT *empirical power) for the various MOIs (Dominant, Multiplicative, Recessive) and significance levels (5%, 1%). *TDT *empirical power is computed using the method we previously published [27].

While the median power difference is approximately 0 for all six categories, we see that there is a pattern associated with disease MOI. That is, for the dominant MOIs, *TDT *tends to have larger power than *TDT-HET *(gray quartile boxes below 0 in Figure 5), while for multiplicative and recessive MOIs, *TDT-HET *tends to have higher power than *TDT *(gray quartile boxes above 0 in Figure 5). The minimum value for power difference of -0.05 occurs for the parameter settings: *ϕ *= 0.05; MOI = Dominant; *R*_2 _= 2.25; DAF = 0.50; *π*_1 _= 0.50; Significance Level = 1%. For these settings, *TDT-HET *empirical power is 0.45, while *TDT *empirical power is 0.50. The maximum value for power difference of 0.17 occurs for the parameter settings: *ϕ *= 0.05; MOI = Recessive; *R*_2 _= 2.25; DAF = 0.25; *π*_1 _= 0.75; Significance Level = 1%. For these settings, *TDT-HET *empirical power is 0.74, while *TDT *empirical power is 0.56.

#### Multi-locus

**Figure 6:**
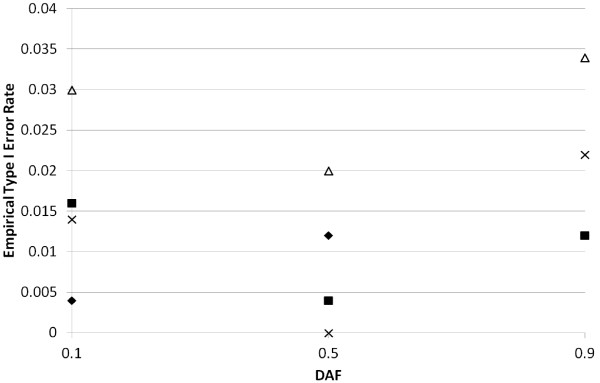
**Empirical type I error rates for *TDT-HET *and *TDT SumStat *statistics**. In this figure, we provide *TDT-HET *and *TDT SumStat *empirical type I error values (GRR *R*_1 _= *R*_2 _= 1.0) for parameter settings listed in Tables 1 and 2. In this figure, solid symbols represent *TDT-HET SumStat *empirical powers; hollow shapes represent *TDT SumStat *empirical powers. More specifically: Solid diamonds = *TDT-HET SumStat *empirical type I error rate at 1% significance level when *π*_1 _= 0.25. Solid squares = *TDT-HET SumStat *empirical type I error rate at 1% significance level when *π*_1 _= 0.75. Hollow diamonds = *TDT SumStat *empirical type I error rate at 1% significance level when *π*_1 _= 0.25. Multiplication signs = *TDT SumStat *empirical type I error rate at 1% significance level when *π*_1 _= 0.75.

**Figure 7:**
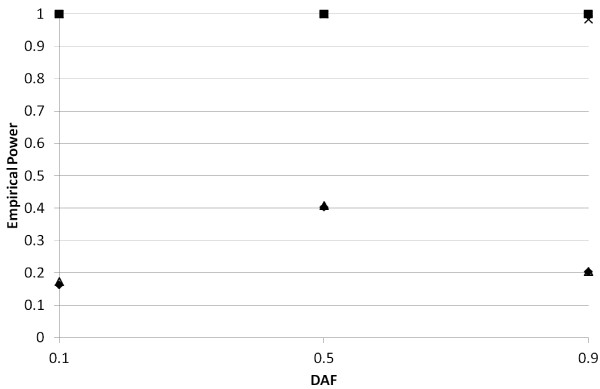
**Empirical powers for *TDT-HET *and *TDT SumStat *statistics when GRR *R***_**1 **_**= 1.5**. Here, we present *TDT-HET *and *TDT SumStat *empirical powers for parameter settings listed in Tables 1 and 2. Solid diamonds = *TDT-HET SumStat *empirical power at 1% significance level when *π*_1 _= 0.25. Solid squares = *TDT-HET SumStat *empirical power at 1% significance level when *π*_1 _= 0.75. Hollow diamonds = *TDT SumStat *empirical power at 1% significance level when *π*_1 _= 0.25. Multiplication signs = *TDT SumStat *empirical power at 1% significance level when *π*_1 _= 0.75.

**Figure 8:**
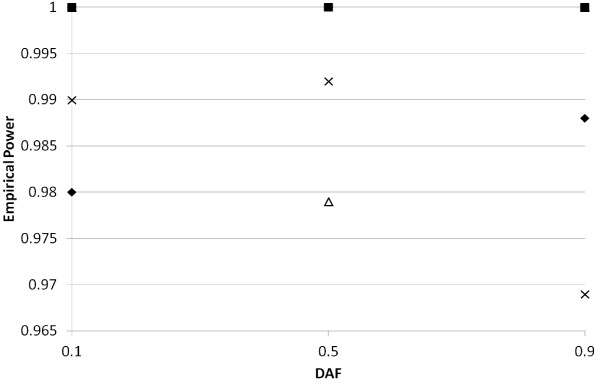
**Empirical powers for *TDT-HET *and *TDT SumStat *statistics when GRR *R***_**1 **_**= 3.0**. Here, we present *TDT-HET *and *TDT SumStat *empirical powers for parameter settings listed in Tables 1 and 2. Solid diamonds = *TDT-HET SumStat *empirical power at 1% significance level when *π*_1 _= 0.25. Solid squares = *TDT-HET SumStat *empirical power at 1% significance level when *π*_1 _= 0.75. Hollow diamonds = *TDT SumStat *empirical power at 1% significance level when *π*_1 _= 0.25. Multiplication signs = *TDT SumStat *empirical power at 1% significance level when *π*_1 _= 0.75.

In Figures 6, 7 and 8, we present *TDT-HET *and *TDT SumStat *empirical power values (type I error rate values in Figure 6) for parameter settings listed in the Methods. The results presented in Figure 6 (*R*_2 _= 1.0) indicate that our *SumStat *statistic appears to maintain the correct type I error rate for all simulation parameters considered, with the exception of the settings: *TDT SumStat Statistic, π*_1 _= 0.25, DAF = 0.90, which gives an empirical type I error rate of 0.03 at the 1% level. According to BINOM, the exact 95% confidence interval for this value does not contain 0.01 (the lower bound of the interval is 0.02). However, all other simulations do contain 0.01 in their 95% confidence intervals.

Regarding empirical power, when *R*_1 _= 1.5 (Figure 7), *TDT-HET *and *TDT *produce nearly identical powers. This can be seen from the fact that the hollow symbols of the *TDT *empirical powers do not seem to appear in Figure 7. The reason is that they are covered by the *TDT-HET *empirical power symbols. When *R*_1 _= 3.0 (Figure 8), *TDT-HET *and *TDT *also produce nearly identical powers. Note that the values on the vertical axis for Figure 8 are much higher than those for Figures 6 and 7. We comment that, when *R*_1 _= 3.0, we have very high power at the 1% significance level even with the proportion of linked trios is low (*π*_1 _= 0.25; diamonds and triangles; Figure 8). This result suggests that genotype relative risk can "trump" locus heterogeneity. We have observed this phenomenon in previous studies, where genotype relative risk is the most significant factor in determining power [76], even in the presence of "missing data" (e.g., misclassification errors).

### Idiopathic Scoliosis Candidate Loci

In Table 3, we present the results of our *TDT-HET *analysis for the five candidate loci mentioned in the Methods section. They are: RS1400180, RS10510181 (both on Chromosome 3), RS11770843 (on Chromosome 7); and RS1040315, RS2222973 (both on Chromosome 21).

**Table 3:** Results of *TDT-HET *analysis on idiopathic scoliosis candidate loci

									PLINK Results				
**Chr**	**Locus**	**BP**	***TDT-HET***	**P-value (Perm)**	$\hat{t}$	$\hat{\pi_{1}}$	***TDT-HET SumStat***	***SumStat *P-value (Perm)**	***TDT***	**P-value (Perm01)**	**OR**	$\hat{t}$	**Max(T) P-value (Perm02)**

**3**	RS1400180	145968	14.78	1 6 × 10^-4^	0.80	0.30	23.93	1 0 × 10^-5^	14.35	2.8 × 10^-4^	1.44	0.59	0.001
**3**	RS10510181	166047	9.15	0.003	0.60	0.77			9.04	0.004	1.37	0.58	0.02
**7**	RS11770843	146426312	18.32	1.0 × 10^-5^	0.26	0.47	NA		17.29	1.6 × 10^-4^	1.56	0.61	3.3 × 10^-4^
**21**	RS1040315	40746722	18.41	2.0 × 10^-5^	0.76	0.38	40.94	0.00	19.10	3.0 × 10^-5^	1.54	0.61	1.2 × 10^-4^
**21**	RS2222973	40755754	22.53	0.00	0.36	0.87			22.25	2.0 × 10^-5^	0.60	0.38	7.0 × 10^-5^

The headings for each of the columns are defined as follows:

Chr = Human chromosome on which locus is located.

Locus = Particular SNP genotyped in idiopathic scoliosis trios.

BP = Base pair position of Locus. This position is based on the human reference sequence (NCBI Build 36.1/HG18).

*TDT-HET *= Value of the *TDT-HET *statistic for particular locus genotype data in idiopathic scoliosis trios.

P-value (Perm) = P-value of corresponding *TDT-HET *statistic, based on 100,000 random permutations. For a description of how the permutation p-value is computed, see Methods, *P-values by permutation.*

$\hat{t}$ = EM-Algorithm estimate of the probability, *t*, that a heterozygous parent transmits a "1" allele.

$\hat{\pi_{1}}$ = EM-Algorithm estimate of the probability, *π*_1_, that a trio is linked to the locus in question.

*TDT-HET SumStat *= ∑_*k *_*TDT-HET *(*k*), where *k *indexes the set of all loci on a chromosome and *TDT-HET *(*k*) is the value of the *TDT-HET *statistic at the particular locus. For example, in Table 3, *k *= 1 or 2, corresponding to locus RS1400180 or RS10510181, respectively. The *TDT-HET *statistic for each locus is 14.78 (*k *= 1) and 9.15 (*k *= 2). Therefore, for Chromosome 3, *TDT-HET SumStat *= 14.78 + 9.15 = 23.93.

*SumStat *P-value (Perm) = Permutation P-value corresponding the *TDT-HET SumStat *value. For a further description, see Methods, *Simulations, Multi-locus*.

(PLINK Results)

*TDT *= Value of the *TDT *statistic as computed by PLINK.

P-value (Perm01) = Permutation p-value computed by PLINK. Purcell et al. [74] label this p-value "Emp1". It is the Point-wise empirical p-value.

OR = Odds Ratio for the disease allele.

$\hat{t}=\frac{T}{T+NT}$ = The maximum likelihood estimate of the probability, *t*, that a heterozygous parent transmits the disease allele. Here, *T *is the number of times a heterozygous parent transmits the disease allele, and *NT *= the number of times a heterozygous parent does not transmit the disease allele. It has been shown that, for the likelihood form of the *TDT*, this value is the maximum likelihood estimate of the transmission probability (see, e.g., [81-83]).

Max(T) P-value (Perm02) = Permutation p-value computed by PLINK that controls the family-wise type I error rate. For more information, see Methods, *Idiopathic Scoliosis Candidate Loci*.

The first thing to notice about these results is that the statistic values are similar. For example, on Chromosome 3, locus RS1400180 has a *TDT-HET *statistic value of 14.78 versus a *TDT *value of 14.35. Similarly, on Chromosome 21, locus RS2222973 has a *TDT-HET *statistic value of 22.53 versus a *TDT *value of 22.25. However, as noted above, the *TDT-HET *statistic does not follow a central chi-squared distribution with 1 degree of freedom under the null hypothesis. For that, we must compare permutation p-values. If we compare the point-wise permutation p-values (P-value (Perm) column for *TDT-HET *and Perm01 column for PLINK *TDT*), we see that, for most loci the permutation p-values are quite similar (same order of magnitude). In fact, according to BINOM, for most of the loci, the exact 95% confidence intervals overlap (full results not shown). The one exception is for locus RS11770843 on Chromosome 7. For this locus, the upper bound of the exact 95% confidence interval of the *TDT-HET *permutation p-value as computed by BINOM is 5.6 × 10^-5^, while the lower bound of the exact 95% confidence interval of the PLINK *TDT *permutation p-value (Perm01) is 9.1 × 10^-5^. This result suggests that, for this marker locus, the *TDT-HET *has slightly more power.

As for the multi-locus results, the situation is quite similar. The Bonferroni corrected minimum p-value of the *TDT-HET SumStat *statistic is 0.00, on Chromosome 21. The upper bound of the exact 95% confidence interval is 3.0 × 10^-5^. The lower bound of the exact 95% confidence interval for the minimum max(T) p-value is 2.8 × 10^-5^, indicating that the p-values overlap. Thus power for each method is equivalent for this data set. While additional studies need to be performed, this result suggests that the *SumStat *method for *TDT-HET *may not be as advantageous when loci are in HWE and/or are in linkage disequilibrium.

If there is no gain in power for the *TDT-HET *method over the standard *TDT *method, what is its utility? We suggest that the value comes from the estimates of the transmission probability, the proportion of linked trios, and most especially, the estimates of the probabilities that each of the trios is linked to a particular locus. Similar information is available for the HLOD statistic in that we may obtain probability estimates that each family is linked to a particular locus [38].

We demonstrate this utility for locus RS2222973, on Chromosome 21. In Table 4, we present the posterior probability estimates ${\hat{\tau }}_{1,abc}^{\left(r\right)}$ that each of the 10 coded trios *abc *(see Table 5) is linked to the locus. We note that in Table 3, the estimate of the overall proportion of linked trios, $\hat{\pi_{1}}$, is 0.87. As Ott points out [32] and [38] (page 224), this value can be used as a cutoff for determining coded trios that are linked to the locus as compared with coded trios that are not. Specifically, if ${\hat{\tau }}_{1,abc}^{\left(r\right)}>\hat{\pi_{1}}$, then we conclude that the coded trio *x*_*abc *_is linked to the locus. Similarly, if ${\hat{\tau }}_{1,abc}^{\left(r\right)}<\hat{\pi_{1}}$, then we conclude that the coded trio *x*_*abc *_is unlinked. The results in Table 4 suggest that the coded trios *x*_000_, *x*_100_, *x*_110_, *x*_201_, *x*_211_, *x*_222 _are linked to locus RS2222973, while the remaining 4 coded trios *x*_101_, *x*_111_, *x*_112_, *x*_212 _are not. Of the coded trios for which at least one parent is heterozygous (i.e., either *a *or *b *equals 1), the linked coded trios are defined by the fact that the affected child always receives the "1" allele from a heterozygous parent. This result suggests that, for this locus, the "1" allele is the risk allele.

**Table 4:** Posterior probability estimates that each coded trio is in linked group for Chromosome 21 Locus RS2222973 in the idiopathic scoliosis data set

**Coded trio *x***_***abc***_	${\hat{\tau }}_{1,abc}^{\left(r\right)}$
000	**0.87**
100	**0.90**
**101**	0.83
110	**0.92**
**111**	0.86
**112**	0.78
201	**0.87**
211	**0.90**
**212**	0.83
222	**0.87**

We indicate in bold the coded trios *x*_*abc *_such that ${\hat{\tau }}_{1,abc}^{\left(r\right)}\geq \hat{\pi_{1}}=0.87$. The value 0.87 comes from Table 3, for locus RS2222973. See Results, *Idiopathic Scoliosis Candidate Loci*, for further discussion of the importance of this inequality.

**Table 5:** Conditional probabilities of mating type and child genotype

Mating type = *i*	**Pr(Mating type = *i***|***D***, **pop = *k*)**	Child genotype	Notation	**Pr(Child genotype|*D***, **Mating type = *i*, pop = *k*) (*t *= 1/2 when *k *= 2)**	Pr(*x*_*abc*_|*D*, pop = *k*)
MM × MM (***i *= 1**)	*μ*_*k*,1_	MM	*x*_222_	1	*μ*_*k*,1_
MM × MNC(***i *= 2**)	*μ*_*k*,2_	MM	*x*_212_	*t*	*μ*_*k*,2 _*t*
MM × MNC(***i *= 2**)	*μ*_*k*,2_	MN	*x*_211_	(1 - *t*)	*μ*_*k*,2 _(1 - *t*)
MM × NN(***i *= 3**)	*μ*_*k*,3_	MN	*x*_201_	1	*μ*_*k*,3_
MN × MN(***i *= 4**)	*μ*_*k*,4_	MM	*x*_112_	*t*^2^	*μ*_*k*,4 _*t*^2^
MN × MN(***i *= 4**)	*μ*_*k*,4_	MN	*x*_111_	2*t*(1 - *t*)	2 *μ*_*k,*4 _*t*(1 - *t*)
MN × MN(***i *= 4**)	*μ*_*k*,4_	NN	*x*_110_	(1 - *t*)^2^	*μ*_*k*,4_(1 - *t*)^2^
MN × NN(***i *= 5**)	*μ*_*k*,5_	MN	*x*_101_	*t*	*μ*_*k*,5 _*t*
MN × NN(***i *= 5**)	*μ*_*k*,5_	NN	*x*_100_	(1 - *t*)	*μ*_*k,5 *_(1 - *t*)
NN × NN(***i *= 6**)	*μ*_*k*,6_	NN	*x*_000_	1	*μ*_*k,*6_

In this table, the high risk allele is M. Also, we define *D *to be the event that the child is affected. Note that 1 ≤ *k *≤ 2. The last column is computed using the definition of conditional probability. Schaid and Sommer [63] also demonstrated this calculation. Note that $\text{Pr}\left(x_{abc}|D,\text{pop}=k\right)=f_{B}\left(x_{abc};{\vec{\theta }}_{k}\right)$. Finally, *t *= *Pr*(heterozygous parent transmits an M allele to an affected child).

## Discussion

In this work, we present a mixture model of linked and unlinked trios and develop a statistical method to estimate the probability *t *that a heterozygous parent transmits the disease allele at a di-allelic locus, as well as the probability ***π***_1 _that any trio is in the linked group. The null hypothesis is that *t *= 0.5. The purpose here is the development of a test, the *TDT-HET*, which extends the classic transmission disequilibrium test (*TDT*) to one that accounts for locus heterogeneity. Our results suggest that use of permutation p-values enable us to correctly maintain correct type I error rates at the 5% and 1% significance levels. Power simulations using disease MOIs suggest that power can be disease model dependent, with the *TDT *being slightly more powerful for dominant MOIs, and the *TDT-HET *being more power for recessive MOIs. Also, we find that our statistic can have high power, even in the presence of locus heterogeneity, when the GRR is larger.

It is interesting to note that the value of the *TDT-HET *statistic and the corresponding permutation p-value appears to be about the same as that of ordinary *TDT *for the Idiopathic scoliosis Candidate Loci data set even though results of the *TDT-HET *analysis suggest that there is locus heterogeneity for several loci. Based on our simulations, we might conjecture that the single-locus MOI for each SNP is multiplicative.

We computed parameters for the situation where linked and unlinked trio types come from populations with different sets of parental mating type frequencies, but apart from determining the *r*^th ^iteration step estimates, we did not investigate this form of the *TDT-HET *statistic further. Given the extensive amount of work already present, we consider this work to be beyond scope of the present manuscript. We plan to follow up this research and report our findings in another manuscript.

As noted in Results, Idiopathic Scoliosis Candidate Loci section, Ott documents that a decision rule for determining whether a particular trio type *x*_*abc *_is linked to a locus is seeing whether the inequality:

(2) $${\hat{\tau }}_{1,abc}^{\left(r\right)}\geq \hat{\pi_{1}},$$

is satisfied. Having said that, Terwilliger and Ott [77] report that, for linkage, the conditional probabilities "...should be taken with a grain of salt, and they cannot ever be validly used to separate families for the remainder of a linkage study. It should be required that any further marker typings be done on all families combined..." Their rationale for this statement is that selectively typing only linked families would introduce bias and increase the type I error rate of the linkage statistic. However, this book was published in 1994, even before the advent of SNPs. We are now producing next generation sequence data, so that the causative variant may well be typed in the first set. It remains an open question whether one can use the parameter estimates ${\hat{\tau }}_{1,abc}^{\left(r\right)}$ to find trios that contain the causative variant(s). We recognize that there are situations where parameter estimation may be quite difficult. Vieland and Logue [78] documented that when the genetic models at linked and unlinked loci differ, maximizing the HLOD yields incorrect parameter estimates. These authors found that the admixture parameter *α *does not even measure the proportion of linked families within the sample, as is commonly supposed.

We conjecture that having additional information on the posterior probabilities ${\hat{\tau }}_{1,abc}^{\left(r\right)}$ may increase the probability of correctly identifying linked trios. One of the advantages of the *TDT-HET *statistic is that it provides estimates that each of the 10 types of trios (Table 5) is linked/unlinked. We can use this information to create a decision rule about whether a particular trio type is linked (i.e., harbors the disease allele). One possible decision rule is the inequality documented by Ott [38] and listed above (2). Ott reports that, for linkage analysis allowing for locus heterogeneity, a decision rule for determining whether a particular family is linked to a locus is checking whether the posterior probability that the family is linked is larger than or equal to the overall estimate of the proportion of linked families. We can extend this rule to our work by making the decision rule be that a trio type *x*_*abc *_is linked to a locus if and only if the inequality is satisfied. Here *r *is the iteration step such that the log-likelihoods are less than the stopping criterion.

This decision rule potentially reduces the number of trios that we need consider when looking for linked trios. We can further reduce the number of trios considered by adding the condition that we only consider trios in which at least one parent is heterozygous. Thus, the two decision rules we consider here for selecting linked trios using the *TDT-HET *statistic are: (i) all trios that satisfy inequality (2); and (ii) all trios for which at least one parent is heterozygous and that also satisfy inequality (2).

For the *TDT *statistic, our analogous decision rules are: (i) all trios; and (ii) all trios for which at least parent is heterozygous.

We plan to perform an extensive analysis to evaluate the empirical probabilities that each statistic can correctly identify linked trios. We can simulate linked and unlinked trios using the method implemented in the FASTSLINK software [79,80]. We can use different genetic model parameter settings, specifically, settings in which the genetic effect is small/large. Since FASTLINK produces pedigree files that indicate which pedigrees are linked or unlinked, we can directly test our decision rules. This is work in progress.

Given that next generation sequencing data applied to families is bound to identify large amounts of locus heterogeneity, any methods that increase the probability of identifying true disease variants should be welcome. We realize that, though, the probabilities of correctly identifying linked trios may be dependent upon the true proportion of linked trios. One way we can reduce heterogeneity is to look at larger family sizes. We plan to apply our statistic to such families and investigate its performance.

## Conclusions

Motivated by the recent work of Zhou and Pan [58], we have developed a *TDT *statistic, *TDT-HET*, that allows for locus heterogeneity among coded trios. This method is an extension of *TDT*, in that our simulation results suggest it has approximately the same power as the original TDT. Results of our simulations suggest that our method maintains correct type I error for the null hypothesis (*R*_1 _= 1.0). Benefits of our method include: estimates of parameters in the presence of heterogeneity (e.g., the proportion of linked coded trios, the posterior probabilities that a particular trio type is linked to a locus), and reasonable power even when the proportion of linked trios is lower. Also, we have extended Hoh, Ott, and colleagues' *SumStat *method to *TDT-HET*. The parameter estimation above, particular, estimation of the probability that a trio is linked will be useful as we enter the age of next-generation sequencing, where one can expect extensive levels of locus heterogeneity given the rare disease frequencies.

## Authors' contributions

DL co-developed the TDT-het statistic, performed a number of analyses and simulations and wrote a significant portion of the manuscript. SB and SJF both helped develop the *TDT-HET *method and provided advice on application of the Expectation-Maximization Algorithm. SS performed the genotyping for the Idiopathic Scoliosis candidate loci samples and, together with CAW, wrote the section describing the Idiopathic Scoliosis candidate loci sample collection. CAW graciously provided samples for the Idiopathic Scoliosis candidate loci example. DG co-developed the *TDT-HET *statistic, wrote software code to compute the statistic, performed a number of simulations and wrote the majority of the manuscript. All authors read and approved the final manuscript.

## Supplementary Material

Additional file 1 **Appendix**. Full details of the derivation of the *TDT-HET *statistic, including notation. Click here for file

## References

[B1] Smith CAB Homogeneity test for linkage data Proc Sec Int Congr Hum Genet 1961 1 212 213

[B2] Smith CAB Testing for heterogeneity of recombination fraction values in human genetics Ann Hum Genet 1963 27 175 182 10.1111/j.1469-1809.1963.tb00210.x 14081488

[B3] Duncan JA Reeves JR Cooke TG BRCA1 and BRCA2 proteins: roles in health and disease Mol Pathol 1998 51 5 237 247 10.1136/mp.51.5.237 PMC395646 10193517

[B4] Hall JM Lee MK Newman B Morrow JE Anderson LA Huey B King MC Linkage of early-onset familial breast cancer to chromosome 17q21 Science 1990 250 4988 1684 1689 10.1126/science.2270482 2270482

[B5] Miki Y Swensen J Shattuck-Eidens D Futreal PA Harshman K Tavtigian S Liu Q Cochran C Bennett LM Ding W Bell R Rosenthal J Hussey C Tran T McClure M Frye C Hattier T Phelps R Haugen-Strano A Katcher H Yakumo K Gholami Z Shaffer D Stone S Bayer S Wray C Bogden R Dayananth P Ward J Tonin P A strong candidate for the breast and ovarian cancer susceptibility gene BRCA1 Science 1994 266 5182 66 71 10.1126/science.7545954 7545954

[B6] Wooster R Neuhausen SL Mangion J Quirk Y Ford D Collins N Nguyen K Seal S Tran T Averill D Fields P Marshall G Narod S Lenoir GM Lynch H Feunteun J Devilee P Cornelisse CJ Menko FH Daly PA Ormiston W McManus R Pye C Lewis CM Cannon-Albright LA Peto J Ponder BAJ Skolnick MH Easton DF Goldgar DE Localization of a breast cancer susceptibility gene, BRCA2, to chromosome 13q12-13 Science 1994 265 5181 2088 2090 10.1126/science.8091231 8091231

[B7] Froguel P Velho G Molecular Genetics of Maturity-onset Diabetes of the Young Trends Endocrinol Metab 1999 10 4 142 146 10.1016/s1043-2760(98)00134-9 10322408

[B8] De Marco EV Gambardella A Annesi F Labate A Carrideo S Forabosco P Civitelli D Candiano IC Tarantino P Annesi G Quattrone A Further evidence of genetic heterogeneity in families with autosomal dominant nocturnal frontal lobe epilepsy Epilepsy Res 2007 74 1 70 73 10.1016/j.eplepsyres.2006.12.006 17324557

[B9] Selkoe DJ Amyloid beta-protein and the genetics of Alzheimer's disease J Biol Chem 1996 271 31 18295 18298 10.1074/jbc.271.31.18295 8756120

[B10] Criswell LA Chen WV Jawaheer D Lum RF Wener MH Gu X Gregersen PK Amos CI Dissecting the heterogeneity of rheumatoid arthritis through linkage analysis of quantitative traits Arthritis Rheum 2007 56 1 58 68 10.1002/art.22325 17195208

[B11] Nystrom-Lahti M Parsons R Sistonen P Pylkkanen L Aaltonen LA Leach FS Hamilton SR Watson P Bronson E Fusaro R Cavalieri J Lynch J Lanspa S Smyrk T Lynch P Drouhard T Kinzler KW Vogelstein B Lynch HT Chapelle Adl Peltomäki P Mismatch repair genes on chromosomes 2p and 3p account for a major share of hereditary nonpolyposis colorectal cancer families evaluable by linkage Am J Hum Genet 1994 55 4 659 665 PMC1918295 7942843

[B12] Kelsell DP Dunlop J Stevens HP Lench NJ Liang JN Parry G Mueller RF Leigh IM Connexin 26 mutations in hereditary non-syndromic sensorineural deafness Nature 1997 387 6628 80 83 10.1038/387080a0 9139825

[B13] Grifa A Wagner CA D'Ambrosio L Melchionda S Bernardi F Lopez-Bigas N Rabionet R Arbones M Monica MD Estivill X Zelante L Lang F Gasparini P Mutations in GJB6 cause nonsyndromic autosomal dominant deafness at DFNA3 locus Nat Genet 1999 23 1 16 18 10.1038/12612 10471490

[B14] Van Laer L Huizing EH Verstreken M van Zuijlen D Wauters JG Bossuyt PJ Van de Heyning P McGuirt WT Smith RJ Willems PJ Legan PK Richardson GP Van Camp G Nonsyndromic hearing impairment is associated with a mutation in DFNA5 Nat Genet 1998 20 2 194 197 10.1038/2503 9771715

[B15] Dryja TP Li T Molecular genetics of retinitis pigmentosa Hum Mol Genet 1995 4 1739 1743 Spec No 10.1093/hmg/4.suppl_1.1739 8541873

[B16] Papaioannou M Chakarova CF Prescott DC Waseem N Theis T Lopez I Gill B Koenekoop RK Bhattacharya SS A new locus (RP31) for autosomal dominant retinitis pigmentosa maps to chromosome 9p Hum Genet 2005 118 3-4 501 503 10.1007/s00439-005-0063-3 16189705

[B17] Tong Z Yang Z Meyer JJ McInnes AW Xue L Azimi AM Baird J Zhao Y Pearson E Wang C Chen Y Zhang K A novel locus for X-linked retinitis pigmentosa Ann Acad Med Singapore 2006 35 7 476 478 16902723

[B18] Huang J Vieland VJ Comparison of 'model-free' and 'model-based' linkage statistics in the presence of locus heterogeneity: single data set and multiple data set applications Hum Hered 2001 51 4 217 225 10.1159/000053345 11287743

[B19] MacLean CJ Ploughman LM Diehl SR Kendler KS A new test for linkage in the presence of locus heterogeneity Am J Hum Genet 1992 50 6 1259 1266 PMC1682581 1598905

[B20] Teare DM Barrett JH Genetic linkage studies The Lancet 2005 366 9490 1036 1044 10.1016/S0140-6736(05)67382-5 16168786

[B21] Vieland VJ Wang K Huang J Power to detect linkage based on multiple sets of data in the presence of locus heterogeneity: comparative evaluation of model-based linkage methods for affected sib pair data Hum Hered 2001 51 4 199 208 10.1159/000053343 11287741

[B22] Wang D Huang J Detecting linkage disequilibrium in the presence of locus heterogeneity Ann Hum Genet 2006 70 Pt 3 397 409 10.1111/j.1529-8817.2005.00229.x 16674561

[B23] Abreu PC Greenberg DA Hodge SE Direct power comparisons between simple LOD scores and NPL scores for linkage analysis in complex diseases Am J Hum Genet 1999 65 3 847 857 10.1086/302536 PMC1377991 10441591

[B24] Abreu PC Hodge SE Greenberg DA Quantification of type I error probabilities for heterogeneity LOD scores Genet Epidemiol 2002 22 2 156 169 10.1002/gepi.0155 11788961

[B25] Falk CT Effect of genetic heterogeneity and assortative mating on linkage analysis: a simulation study Am J Hum Genet 1997 61 5 1169 1178 10.1086/301591 PMC1716020 9345086

[B26] Chiano MN Yates JR Bootstrapping in human genetic linkage Ann Hum Genet 1994 58 Pt 2 129 143 10.1111/j.1469-1809.1994.tb01882.x 7979157

[B27] Chen C Yang G Buyske S Matise T Finch SJ Gordon D Transmission disequilibrium test power and sample size in the presence of locus heterogeneity Stat Appl Genet Mol Biol 2009 8 1 44 Article 10.2202/1544-6115.1501 19883370

[B28] Morton NE The detection and estimation of linkage between the genes for elliptocytosis and the Rh blood type Am J Hum Genet 1956 8 80 96 PMC1716663 13313518

[B29] Risch N A new statistical test for linkage heterogeneity Am J Hum Genet 1988 42 2 353 364 PMC1715251 3341384

[B30] Goldstein DR A combined test of linkage heterogeneity Am J Hum Genet 1994 55 4 841 848 PMC1918301 7942861

[B31] Hodge SE Anderson CE Neiswanger K Sparkes RS Rimoin DL The search for heterogeneity in insulin-dependent diabetes mellitus (IDDM): Linkage studies, two-locus models, and genetic heterogeneity Am J Hum Genet 1983 35 1139 1155 PMC1685977 6580815

[B32] Ott J Linkage analysis and family classification under heterogeneity Ann Hum Genet 1983 47 311 320 10.1111/j.1469-1809.1983.tb01001.x 6651220

[B33] Risch N Baron M X-linkage and genetic heterogeneity in bipolar-related major affective illness: reanalysis of linkage data Ann Hum Genet 1982 46 Pt 2 153 166 10.1111/j.1469-1809.1982.tb00706.x 6985470

[B34] Ott J Counting methods (EM algorithm) in human pedigree analysis: linkage and segregation analysis Ann Hum Genet 1977 40 4 443 454 879713

[B35] Faraway JJ Distribution of the admixture test for the detection of linkage under heterogeneity Genet Epidemiol 1993 10 1 75 83 10.1002/gepi.1370100108 8472936

[B36] Ott J Strategies for characterizing highly polymorphic markers in human gene mapping Am J Hum Genet 1992 51 2 283 290 PMC1682689 1642229

[B37] Morton NE Sequential tests for the detection of linkage Am J Hum Genet 1955 7 3 277 318 PMC1716611 13258560

[B38] Ott J Analysis of Human Genetic Linkage 1999 Third Baltimore, MD: The John Hopkins University Press

[B39] Bhat A Heath SC Ott J Heterogeneity for multiple disease loci in linkage analysis Hum Hered 1999 49 4 229 231 10.1159/000022879 10436385

[B40] Yang X Wang K Huang J Vieland VJ Genome-wide linkage analysis of blood pressure under locus heterogeneity BMC Genet 2003 4 Suppl 1 S78 10.1186/1471-2156-4-S1-S78 PMC1866517 14975146

[B41] Knight J North BV Sham PC Curtis D Mapping loci influencing blood pressure in the Framingham pedigrees using model-free LOD score analysis of a quantitative trait BMC Genet 2003 4 Suppl 1 S74 10.1186/1471-2156-4-S1-S74 PMC1866513 14975142

[B42] Ekstrom CT Dalgaard P Linkage analysis of quantitative trait loci in the presence of heterogeneity Hum Hered 2003 55 1 16 26 10.1159/000071806 12890922

[B43] Wang K Peng Y Quantitative-trait-locus mapping in the presence of locus heterogeneity Ann Hum Genet 2006 70 Pt 6 882 892 10.1111/j.1469-1809.2006.00277.x 17044863

[B44] Lazarsfeld PFWHN Latent Structure Analysis 1968 Boston: Houghton Mifflin

[B45] Holliday EG McLean DE Nyholt DR Mowry BJ Susceptibility locus on chromosome 1q23-25 for a schizophrenia subtype resembling deficit schizophrenia identified by latent class analysis Arch Gen Psychiatry 2009 66 10 1058 1067 10.1001/archgenpsychiatry.2009.136 19805696

[B46] Todd RD Rasmussen ER Neuman RJ Reich W Hudziak JJ Bucholz KK Madden PA Heath A Familiality and heritability of subtypes of attention deficit hyperactivity disorder in a population sample of adolescent female twins Am J Psychiatry 2001 158 11 1891 1898 10.1176/appi.ajp.158.11.1891 11691697

[B47] Bureau A Croteau J Tayeb A Merette C Labbe A Latent class model with familial dependence to address heterogeneity in complex diseases: adapting the approach to family-based association studies Genet Epidemiol 2011 35 3 182 189 10.1002/gepi.20566 PMC4000257 21308764

[B48] Derks EM Allardyce J Boks MP Ophoff RA Improvement of phenotyping in genome wide association studies on schizophrenia: an application of latent class factor analysis Schizophrenia Research 2010 117 2-3 184 185

[B49] Macgregor S Craddock N Holmans PA Use of phenotypic covariates in association analysis by sequential addition of cases Eur J Hum Genet 2006 14 5 529 534 10.1038/sj.ejhg.5201604 16538225

[B50] Qin X Hauser ER Schmidt S Ordered subset analysis for case-control studies Genet Epidemiol 2010 34 5 407 417 10.1002/gepi.20489 PMC2937265 20568256

[B51] Perdry H Maher BS Babron MC McHenry T Clerget-Darpoux F Marazita ML An ordered subset approach to including covariates in the transmission disequilibrium test BMC Proc 2007 1 Suppl 1 S77 10.1186/1753-6561-1-s1-s77 PMC2367525 18466579

[B52] Spielman RS McGinnis RE Ewens WJ Transmission test for linkage disequilibrium: the insulin gene region and insulin-dependent diabetes mellitus (IDDM) Am J Hum Genet 1993 52 3 506 516 PMC1682161 8447318

[B53] Chung RH Schmidt S Martin ER Hauser ER Ordered-subset analysis (OSA) for family-based association mapping of complex traits Genet Epidemiol 2008 32 7 627 637 10.1002/gepi.20340 PMC2582061 18473393

[B54] Martin ER Bass MP Hauser ER Kaplan NL Accounting for linkage in family-based tests of association with missing parental genotypes Am J Hum Genet 2003 73 5 1016 1026 10.1086/378779 PMC1180482 14551902

[B55] Yang X Huang J Logue MW Vieland VJ The posterior probability of linkage allowing for linkage disequilibrium and a new estimate of disequilibrium between a trait and a marker Hum Hered 2005 59 4 210 219 10.1159/000086699 16015031

[B56] Huang Y Vieland VJ Association statistics under the PPL framework Genet Epidemiol 2010 34 8 835 845 10.1002/gepi.20537 21058335

[B57] Schmidt S Schmidt MA Qin X Martin ER Hauser ER Increased efficiency of case-control association analysis by using allele-sharing and covariate information Hum Hered 2008 65 3 154 165 10.1159/000109732 PMC2709298 17934318

[B58] Zhou H Pan W Binomial mixture model-based association tests under genetic heterogeneity Ann Hum Genet 2009 73 Pt 6 614 630 10.1111/j.1469-1809.2009.00542.x PMC3354651 19725835

[B59] Hoh J Ott J A train of thoughts on gene mapping Theor Popul Biol 2001 60 3 149 153 10.1006/tpbi.2001.1536 11855949

[B60] Hoh J Ott J Mathematical multi-locus approaches to localizing complex human trait genes Nat Rev Genet 2003 4 9 701 709 10.1038/nrg1155 12951571

[B61] Hoh J Ott J Genetic dissection of diseases: design and methods Curr Opin Genet Dev 2004 14 3 229 232 10.1016/j.gde.2004.04.006 15172663

[B62] Hoh J Wille A Ott J Trimming, weighting, and grouping SNPs in human case-control association studies Genome Res 2001 11 12 2115 2119 10.1101/gr.204001 PMC311222 11731502

[B63] Schaid DJ Sommer SS Genotype relative risks: methods for design and analysis of candidate-gene association studies Am J Hum Genet 1993 53 5 1114 1126 PMC1682319 8213835

[B64] Weinberg CR Allowing for missing parents in genetic studies of case-parent triads Am J Hum Genet 1999 64 4 1186 1193 10.1086/302337 PMC1377843 10090904

[B65] Weinberg CR Wilcox AJ Lie RT A log-linear approach to case-parent-triad data: assessing effects of disease genes that act either directly or through maternal effects and that may be subject to parental imprinting Am J Hum Genet 1998 62 4 969 978 10.1086/301802 PMC1377041 9529360

[B66] Kruglyak L Daly MJ Reeve-Daly MP Lander ES Parametric and nonparametric linkage analysis: a unified multipoint approach Am J Hum Genet 1996 58 6 1347 1363 PMC1915045 8651312

[B67] Sobel E Lange K Descent graphs in pedigree analysis: applications to haplotyping, location scores, and marker-sharing statistics Am J Hum Genet 1996 58 6 1323 1337 PMC1915074 8651310

[B68] O'Connell JR Weeks DE The VITESSE algorithm for rapid exact multilocus linkage analysis via genotype set-recoding and fuzzy inheritance Nat Genet 1995 11 4 402 408 10.1038/ng1295-402 7493020

[B69] Abecasis GR Cherny SS Cookson WO Cardon LR Merlin--rapid analysis of dense genetic maps using sparse gene flow trees Nat Genet 2002 30 1 97 101 10.1038/ng786 11731797

[B70] Dempster A Laird N Rubin D Maximum likelihood from incomplete data via the EM algorithm J R Stat Soc Ser B 1977 39 1 38

[B71] Sharma S Gao X Londono D Devroy SE Mauldin KN Frankel JT Brandon JM Zhang D Li QZ Dobbs MB Gurnett CA Grant SF Hakonarson H Dormans JP Herring JA Gordon D Wise CA Genome-wide association studies of adolescent idiopathic scoliosis suggest candidate susceptibility genes Hum Mol Genet 20 7 1456 1466 10.1093/hmg/ddq571 PMC3049353 21216876

[B72] Wise CA Gao X Shoemaker S Gordon D Herring JA Understanding genetic factors in idiopathic scoliosis, a complex disease of childhood Current genomics 2008 9 1 51 59 10.2174/138920208783884874 PMC2674301 19424484

[B73] Nelson LM Kenneth W Genetic Markers of Chromosome 7 Associated With Scoliosis And Use Thereof. In., vol. WO/2008/033813 2008 Switzerland: World Intellectual Property Organization

[B74] Purcell S Neale B Todd-Brown K Thomas L Ferreira MA Bender D Maller J Sklar P de Bakker PI Daly MJ Sham PC PLINK: a tool set for whole-genome association and population- based linkage analyses Am J Hum Genet 2007 81 3 559 575 10.1086/519795 PMC1950838 17701901

[B75] Tukey JW Exploratory Data Analysis 1977 Upper Saddle River, NJ: Pearson Education - Addison Wesley

[B76] Ji F Yang Y Haynes C Finch SJ Gordon D Computing asymptotic power and sample size for case-control genetic association studies in the presence of phenotype and/or genotype misclassification errors Stat Appl Genet Mol Biol 2005 4 37 Article 10.2202/1544-6115.1184 16646856

[B77] Terwilliger JD Ott J Handbook of Human Genetic Linkage 1994 Baltimore: Johns Hopkins University Press

[B78] Vieland VJ Logue M HLODs, trait models, and ascertainment: implications of admixture for parameter estimation and linkage detection Hum Hered 2002 53 1 23 35 10.1159/000048601 11901268

[B79] Ott J Computer-simulation methods in human linkage analysis Proceedings of the National Academy of Sciences of the United States of America 1989 86 11 4175 4178 10.1073/pnas.86.11.4175 PMC287412 2726769

[B80] Weeks DE Ott J Lathrop GM SLINK: a general simulation program for linkage analysis Am J Hum Genet 1990 47 A204

[B81] Abel L Muller-Myhsok B Maximum-likelihood expression of the transmission/disequilibrium test and power considerations Am J Hum Genet 1998 63 2 664 667 10.1086/301975 PMC1377316 9683606

[B82] Gordon D Heath SC Liu X Ott J A transmission/disequilibrium test that allows for genotyping errors in the analysis of single-nucleotide polymorphism data Am J Hum Genet 2001 69 2 371 380 10.1086/321981 PMC1235309 11443542

[B83] Tu IP Whittemore AS Power of association and linkage tests when the disease alleles are unobserved Am J Hum Genet 1999 64 2 641 649 10.1086/302253 PMC1377775 9973303

