# Supplementary material for: TDT-HET: A new transmission disequilibrium test that incorporates locus heterogeneity into the analysis of family-based association data
Source: BMC Bioinformatics. 2012 Jan 20;13:13. doi: 10.1186/1471-2105-13-13 (PMC3292499; doi:10.1186/1471-2105-13-13)
Supplement: Additional file 1 — Appendix. Full details of the derivation of the TDT-HET statistic, including notation. [file 1471-2105-13-13-S1.PDF]

APPENDIX

In this section, we present the full details of the *TDT-HET* statistic, including the derivation of log-likelihoods.

Notation

M = The disease allele at the putative disease SNP locus.

N = The non-disease allele at the putative disease SNP locus.

x_{abc} = The trio where parent 1, parent 2, and affected child have *a*, *b*, and *c* copies of the M allele at the putative disease locus (range for all copies: 0 - 2). For example, x_{222} is the trio with mating type MM × MM and affected child genotype MM. **NOTE:** Throughout this work, we will use the notation *abc* interchangeably with x_{abc}.

n_{abc} = The number of trios x_{abc} in the sample.

n = The total number of trios in the study.

D = Event that the child in a trio is affected.

t = Pr(heterozygous parent transmits M allele to affected offspring). In this work, the null hypothesis, H_0, is $t = 0.5$. The alternative hypothesis, H_1, is $t \neq 0.5$.

$\mu_{k,i} = \Pr(\text{Mating type } = i|D, \text{population } = k)$ = probability that the mating type is i given that the child is affected and the trio comes from the kth population, $1 \le k \le 2$. Throughout this work, we shall use the notation $k = 1$ to indicate that the trio is in the linked population $(t \neq 0.5)$ and $k = 2$ to indicate that the trio is in the unlinked population $(t = 0.5)$. Similar to Schaid and Sommer [1], we consider 6 mating types in this work. We recognize that other models, such as those considered by Weinberg and colleagues [2,3], require more than six mating type frequencies. We conjecture that our work extends to such situations.

$\pi_1 = \Pr$(trio is linked to trait locus) = $\Pr(t \neq 0.5)$. In this work we specify that t is the same for all linked trios. This specification is also made for the recombination fraction in some tests of linkage allowing for heterogeneity (see, e.g., work by C. A. B. Smith [4,5] and Ott [6], specifically the method implemented in programs such as HOMOG [6], GENEHUNTER [7], SIMWALK2 [8], VITESSE [9], MERLIN [10], and other programs).

$\pi_2 = 1 - \pi_1$.

$\hat{x}$ = Maximum likelihood estimate (MLE) of the parameter x. This MLE is determined by means of the EM algorithm.

C = A penalty used to stabilize the parameter estimate π_1 in the log-likelihood equation for the true data (see Equation (1)).

$z_{k,j}$ = The indicator variable for population k and trio x_j, where the subscript j indicates the jth trio in the sample.

$f(x_j;\overrightarrow{\theta_k})$= Probability density function of the trio x_j and the vector of parameters $\overrightarrow{\theta_k}, 1 \leq k \leq 2$. The values of this function are provided in Table 1 (right-most column).

$\overrightarrow{\theta_k}, 1 \leq k \leq 2$ = Vector of parameters for the probability density functions $f(x_j;\overrightarrow{\theta_k})$. We consider two scenarios in this work: (1) All trios are drawn from a population with the same set of mating type frequencies; and (2) The linked and the unlinked sets of trios are drawn from populations in which the set of mating type frequencies differ by population. Under scenario (1), we have $\mu_{1,i} = \mu_{2,i} = \mu_i$ for all i, $\overrightarrow{\theta_1} = (t, \pi_1, \mu_1, \mu_2, \mu_3, \mu_4, \mu_5, \mu_6)$, and $\overrightarrow{\theta_2} = (\frac{1}{2}, \pi_2, \mu_1, \mu_2, \mu_3, \mu_4, \mu_5, \mu_6)$. Under scenario (2), each mating type is different for the linked and the unlinked sub-populations. It follows that $\mu_{1,i} \neq \mu_{2,i}$ for at least one i, $\overrightarrow{\theta_1} = (t, \pi_1, \mu_{1,1}, \mu_{1,2}, \mu_{1,3}, \mu_{1,4}, \mu_{1,5}, \mu_{1,6})$ and $\overrightarrow{\theta_2} = (\frac{1}{2}, \pi_2, \mu_{2,1}, \mu_{2,2}, \mu_{2,3}, \mu_{2,4}, \mu_{2,5}, \mu_{2,6})$. We comment that the arrow (⟶) over each θ indicates that the term is a vector of parameters, not estimates of the parameters.

$\vec{a}$ = Starting vector of parameters in $\vec{\theta}_k$ for EM algorithm under H_0 and H_1.

E = Maximum number of steps for EM algorithm per random starting vector.

S = Maximum number of random starting vectors.

$\log\left(L_0^{r,\vec{a}}\right)$ = The log-likelihood under the null (t = 0.5) of the rth iteration step of the EM algorithm when the random starting vector is $\vec{a}$.

$r(\vec{a}) = \min(r, E)$, where E is defined above, and r is defined as the minimum integer to satisfy:

$$\left|\log\left(L_0^{r,\vec{a}}\right) - \log\left(L_0^{r-1,\vec{a}}\right)\right| < \varepsilon,$$

for a pre-specified tolerance ε. Note that, for some circumstances, r may not exist. For these circumstances, we define $r(\vec{a}) = E$.

$\log\left(\widehat{L_0}\right) = \max_{\vec{a}} \log\left(L_0^{r(\vec{a}),\vec{a}}\right)$, the maximum log-likelihood of the data under the null-hypothesis.

$\log\left(\widehat{L_1}\right)$ = The maximum log-likelihood of the data under the alternative hypothesis. It is computed in a similar fashion to $\log\left(\widehat{L_0}\right)$ with the exception (as noted above: definition of $\vec{a}$ above) that the random starting vectors $\vec{a}$ use the $r(\vec{a})^{\text{th}}$ step estimates of $\overrightarrow{\theta_2}$ from $\log\left(\widehat{L_0}\right)$ as fixed coordinates.

$\tau_{k,j}^{(r)} = r^{\text{th}}$ iteration step estimate that the jth trio is in the kth population, $k = 1,2$. Without loss of clarity, we will use sometimes write $\tau_{k,abc}^{(r)}$, where abc refers to the trio x_{abc} (see above).

Log-likelihood of true data

Using the work of Zhou and Pan [11] ,we write the log-likelihood of the true data as:

$$\log(L_c) = \sum_{k=1}^{2} \sum_{j=1}^{n} z_{k,j} \left[\log(\pi_k) + \log\left(f\left(x_j; \overrightarrow{\theta_k}\right)\right)\right] + C\, log(\pi_1), \quad (1)$$

where C is a penalty used to stabilize the parameter estimate π_1, and $z_{k,j}$ is the indicator variable for population k and trio x_j. That is, if trio x_j is in the kth population, then $z_{k,j}$ is 1; otherwise it is 0. Note that, by this equation, there are n trios in our data set. Since we do not know the values of $z_{k,j}$, we use the method of Expectation-Maximization [12] to estimate them, the mixing proportions π_k, and the coordinates of the vectors $\overrightarrow{\theta_k}, 1 \leq k \leq 2$.

All trios drawn from a population with the same set of mating type frequencies

In this situation, as noted above, each mating type is equal for the linked sub-population and the unlinked population. It follows that $\mu_{1,i} = \mu_{2,i} = \mu_i$ for all i, and $\overrightarrow{\theta_1} = (t, \pi_1, \mu_1, \mu_2, \mu_3, \mu_4, \mu_5, \mu_6)$ and $\overrightarrow{\theta_2} = (\frac{1}{2}, \pi_2, \mu_1, \mu_2, \mu_3, \mu_4, \mu_5, \mu_6)$. To compute estimates for these parameters, we use the EM method.

Linked set of trios and unlinked set of trios drawn from populations in which the set of mating type frequencies differ by population

In this situation, each mating type is different for the linked and the unlinked sub-populations. It follows that $\mu_{1,i} \neq \mu_{2,i}$ for at least one i, and $\overrightarrow{\theta_1} = (t, \pi_1, \mu_{1,1}, \mu_{1,2}, \mu_{1,3}, \mu_{1,4}, \mu_{1,5}, \mu_{1,6})$ and $\overrightarrow{\theta_2} = (\frac{1}{2}, \pi_2, \mu_{2,1}, \mu_{2,2}, \mu_{2,3}, \mu_{2,4}, \mu_{2,5}, \mu_{2,6})$. Note that, from Table 1, we have $\sum_{i=1}^{6} \mu_{k,i} = 1, 1 \leq k \leq 2$. As above, to compute estimates for these parameters, we use the EM method.

Log-likelihood of observed data

We can write the log-likelihood of the observed data under the alternative hypothesis as:

$$\log(L_1) = \sum_{j=1}^{n} \log\left(\Pr(x_j)\right) =$$

$$\sum_{j=1}^{n} \log(\Pr(x_j | x_j \text{ in linked sub-population}) \times \Pr(x_j \text{ in linked sub-population})$$

$$+\Pr(x_j | x_j \text{ in unlinked sub-population}) \times \Pr(x_j \text{ in unlinked sub-population}))$$

$$= \sum_{j=1}^{n} \log\left(\hat{\pi}_1 f\left(x_j; \overrightarrow{\theta_1}\right) + \hat{\pi}_2 f\left(x_j; \overrightarrow{\theta_2}\right)\right).$$

The values $f\left(x_j; \overrightarrow{\theta_1}\right)$ and $f\left(x_j; \overrightarrow{\theta_2}\right)$ are provided in Table 1. Note that the null hypothesis can be written as either: $\pi_1 = 0$ or $t = 0.5$ in $\vec{\theta}_1$, in which case the log-likelihood becomes:

$$\log(L_0) = \sum_{j=1}^{n} \log\left(f\left(x_j; \overrightarrow{\theta_2}\right)\right).$$

This situation, in which two different equations lead to the null scenario, also occurs in linkage analysis with the heterogeneity LOD (HLOD or H-LOD) statistic [13,14]. Under such a mixture model, the asymptotic null can be difficult to determine. As documented by Huang and Vieland [15], the asymptotic null distribution of the HLOD statistic depends on the assumed genetic model for the trait. To address this difficulty, we compute p-values by permutation. Zhou and Pan computed p-values for their statistic using permutation as well [11].

P-values determined by permutation

We create each replicate of permuted data as follows: in a given trio, the affected child's genotype is replaced by the genotype generated using a transmission probability of $t = 0.5$ for each heterozygous parent. That is, each heterozygous parent transmits the M allele with probability 50% and the N allele, also with probability 50%. For example, if a trio's genotypes are: MN, NN, and MN for parent 1, parent 2, and affected child, respectively, then the possible permuted genotypes for the trio are: MN, NN, MN and MN, NN, NN, each occurring with 50%

probability. This permutation method is similar to that implemented in the PLINK software program [16].

TDT-HET **statistic**

The *TDT-HET* statistic is given by the formula:

$$TDT\text{-}HET = 2 \times \left[\log\left(\widehat{L_1}\right) - \log\left(\widehat{L_0}\right)\right]. \qquad (2)$$

We define $\log\left(\widehat{L_0}\right)$ and $\log\left(\widehat{L_1}\right)$ as follows. Let $\log\left(L_0^{r,\vec{a}}\right)$ be the log-likelihood under the null (t = 0.5) of the rth-step of the EM algorithm when the random starting vector is $\vec{a}$. We define:

$$\log\left(\widehat{L_0}\right) = \max_{\vec{a}} \log\left(L_0^{r(\vec{a}),\vec{a}}\right),$$

where (see below) $r(\vec{a}) = \min(r, E)$, and r is defined as the minimum integer to satisfy:

$$\left|\log\left(L_0^{r,\vec{a}}\right) - \log\left(L_0^{r-1,\vec{a}}\right)\right| < \varepsilon,$$

for a pre-specified tolerance ε.

Throughout this work, the starting vector $\vec{a}$ under H_1 will use those values in $\overrightarrow{\theta_2}$ maximized under H_0. For example, when the linked and unlinked trios come from the same population, it follows that $\mu_{1,i} = \mu_{2,i} = \mu_i$ for all i, $\overrightarrow{\theta_1} = (t, \pi_1, \mu_1, \mu_2, \mu_3, \mu_4, \mu_5, \mu_6)$, $\overrightarrow{\theta_2} = (\frac{1}{2}, \pi_2, \mu_1, \mu_2, \mu_3, \mu_4, \mu_5, \mu_6)$, and the maximum likelihood estimates of μ_i are determined by the counting method; that is, they remain constant throughout each step of the EM algorithm. Hence, we can rewrite the parameter vectors as: $\overrightarrow{\theta_1} = (t, \pi_1)$ and $\overrightarrow{\theta_2} = (\pi_2)$. First, we find a maximum likelihood estimate for π_2 (denoted $\widehat{\pi_2}$) by applying the EM algorithm to the data under H_0, considering multiple starting values $\vec{a}$ for π_2. Next, we determine a maximum

likelihood estimate for t under H_1 by using multiple starting vectors $\vec{a} = (t, \widehat{\pi_1})$, where $\widehat{\pi_1} = 1 - \widehat{\pi_2}$. That is, each random starting vector $\vec{a}$ under H_1 consists of a random starting value t.

TDT-HET Statistic parameter estimates

Here, we present the rth iteration step parameters in the vectors $\vec{\theta}_k$ for the *TDT-HET* statistic under different scenarios (All trios drawn from common population, Linked set of trios and unlinked set of trios drawn from different populations).

All trios drawn from a population with one set of parental mating types

t Parameter

We can show that the rth iteration step of the transmission probability parameter t is given by:

$$t^{(r)} = \frac{A^{(r)}}{A^{(r)}+B^{(r)}}, \ (3)$$

where

$$A^{(r)} = \mathrm{n}_{212}\tau_{1,212}^{(r)} + 2\mathrm{n}_{112}\tau_{1,112}^{(r)} + \mathrm{n}_{111}\tau_{1,111}^{(r)} + \mathrm{n}_{101}\tau_{1,101}^{(r)},$$

and

$$B^{(r)} = \mathrm{n}_{211}\tau_{1,211}^{(r)} + \mathrm{n}_{111}\tau_{1,111}^{(r)} + 2\mathrm{n}_{110}\tau_{1,110}^{(r)} + \mathrm{n}_{100}\tau_{1,100}^{(r)}.$$

Note that, if π_2 equals 0, so that all the trios are linked $\left(t \neq \frac{1}{2}\right)$, then $\tau_{1,abc}^{(r)}$ is 1 for all trios, and the formula (3) reduces to the quotient of the number of heterozygous parents that transmit the M allele divided by the number of heterozygous parents in the sample. Several authors [17-19] have shown that the quotient is the maximum likelihood estimate of t.

$\boldsymbol{\pi_1}$ Parameter

Our solution for π_1 is given by:

$$\pi_1^{(r)} = \frac{\sum_{abc} n_{abc} \tau_{1,abc}^{(r)} + C}{n + C}, \quad (4)$$

where each of the parameters has been defined in the Methods section (Notation). We comment that equation (4) is virtually identical to the solution provided by Zhou and Pan [11], with the exception that the posterior probabilities $\tau_{1,abc}^{(r)}$ in this work differ from their probabilities.

μ_i Parameters

Our work in the Appendix indicates that the values of these parameters are given by:

$$\begin{aligned}
\mu_1 &= \frac{n_{222}}{n}, \\
\mu_2 &= \frac{n_{212} + n_{211}}{n}, \\
\mu_3 &= \frac{n_{201}}{n}, \\
\mu_4 &= \frac{n_{112} + n_{111} + n_{110}}{n}, \\
\mu_5 &= \frac{n_{101} + n_{100}}{n}, \\
\mu_6 &= \frac{n_{000}}{n}.
\end{aligned} \quad (5)$$

Another way of saying this is that each μ_i is the proportion of trios in the total sample that have mating type *i* (Table 1). Notice that each of these parameters remains constant over all iterations.

Linked set of trios and unlinked set of trios drawn from populations in which the mating type frequencies differ

In this situation, each mating type is different for the linked and the unlinked sub-populations. It follows that $\mu_{1,i} \neq \mu_{2,i}$ for at least one i, and $\vec{\theta}_1 = (t, \pi_1, \mu_{1,1}, \mu_{1,2}, \mu_{1,3}, \mu_{1,4}, \mu_{1,5}, \mu_{1,6})$ and

$\vec{\theta}_2 = (\frac{1}{2}, \pi_2, \mu_{2,1}, \mu_{2,2}, \mu_{2,3}, \mu_{2,4}, \mu_{2,5}, \mu_{2,6})$. Note that, from Table 1, we have $\sum_{i=1}^{6} \mu_{k,i} = 1, 1 \leq k \leq 2$.

$\mu_{k,i}$ Parameters

If we define:

$$n_1^{(r)} = n_{222}\tau_{1,222}^{(r)} + n_{212}\tau_{1,212}^{(r)} + n_{211}\tau_{1,211}^{(r)} + n_{201}\tau_{1,201}^{(r)} + n_{112}\tau_{1,112}^{(r)} + n_{111}\tau_{1,111}^{(r)} + n_{110}\tau_{1,110}^{(r)} + n_{101}\tau_{1,101}^{(r)} + n_{100}\tau_{1,100}^{(r)} + n_{000}\tau_{1,000}^{(r)}$$

we can show (Appendix) that:

$$\mu_{1,1} = \frac{n_{222}\tau_{1,222}^{(r)}}{n_1^{(r)}},$$

$$\mu_{1,2} = \frac{n_{212}\tau_{1,212}^{(r)} + n_{211}\tau_{1,211}^{(r)}}{n_1^{(r)}},$$

$$\mu_{1,3} = \frac{n_{201}\tau_{1,201}^{(r)}}{n_1^{(r)}},$$

$$\mu_{1,4} = \frac{n_{112}\tau_{1,112}^{(r)} + n_{111}\tau_{1,111}^{(r)} + n_{110}\tau_{1,110}^{(r)}}{n_1^{(r)}}, \text{(6a)}$$

$$\mu_{1,5} = \frac{n_{101}\tau_{1,101}^{(r)} + n_{100}\tau_{1,100}^{(r)}}{n_1^{(r)}},$$

$$\mu_{1,6} = \frac{n_{000}\tau_{1,000}^{(r)}}{n_1^{(r)}}.$$

For k =2, one can show that the corresponding mating types are:

$$\mu_{2,1} = \frac{n_{222}\tau_{2,222}^{(r)}}{n_2^{(r)}},$$

$$\mu_{2,2} = \frac{n_{212}\tau_{2,212}^{(r)} + n_{211}\tau_{2,211}^{(r)}}{n_2^{(r)}},$$

$$\mu_{2,3} = \frac{n_{201}\tau_{2,201}^{(r)}}{n_2^{(r)}},$$

$$\mu_{2,4} = \frac{n_{112}\tau_{2,112}^{(r)} + n_{111}\tau_{2,111}^{(r)} + n_{110}\tau_{2,110}^{(r)}}{n_2^{(r)}}, \text{(6b)}$$

$$\mu_{2,5} = \frac{n_{101}\tau_{2,101}^{(r)} + n_{100}\tau_{2,100}^{(r)}}{n_2^{(r)}},$$

$$\mu_{2,6} = \frac{n_{000}\tau_{2,000}^{(r)}}{n_2^{(r)}},$$

where

$$n_2^{(r)} = n_{222}\tau_{2,222}^{(r)} + n_{212}\tau_{2,212}^{(r)} + n_{211}\tau_{2,211}^{(r)} + n_{201}\tau_{2,201}^{(r)} +$$

$$n_{112}\tau_{2,112}^{(r)} + n_{111}\tau_{2,111}^{(r)} + n_{110}\tau_{2,110}^{(r)} + n_{101}\tau_{2,101}^{(r)} + n_{100}\tau_{2,100}^{(r)} + n_{000}\tau_{2,000}^{(r)}$$

analogous to $n_1^{(r)}$ defined above.

We note that the EM estimate $t^{(r)}$ will not change, nor will the estimates ${\pi_k}^{(r)}$. Our reasoning is presented in the Appendix.

References

1. Schaid DJ, Sommer SS. Genotype relative risks: methods for design and analysis of candidate-gene association studies. *Am J Hum Genet.* Nov 1993;53(5):1114-1126.

2. Weinberg CR. Allowing for missing parents in genetic studies of case-parent triads. *Am J Hum Genet.* Apr 1999;64(4):1186-1193.
3. Weinberg CR, Wilcox AJ, Lie RT. A log-linear approach to case-parent-triad data: assessing effects of disease genes that act either directly or through maternal effects and that may be subject to parental imprinting. *Am J Hum Genet.* Apr 1998;62(4):969-978.
4. Smith CAB. Homogeneity test for linkage data. *Proc Sec Int Congr Hum Genet.* 1961;1:212-213.
5. Smith CAB. Testing for heterogeneity of recombination fraction values in human genetics. *Ann Hum Genet.* 1963;27:175-182.
6. Ott J. *Analysis of Human Genetic Linkage.* Baltimore: Johns Hopkins University Press; 1999.
7. Kruglyak L, Daly MJ, Reeve-Daly MP, Lander ES. Parametric and nonparametric linkage analysis: a unified multipoint approach. *Am J Hum Genet.* Jun 1996;58(6):1347-1363.
8. Sobel E, Lange K. Descent graphs in pedigree analysis: applications to haplotyping, location scores, and marker-sharing statistics. *Am J Hum Genet.* Jun 1996;58(6):1323-1337.
9. O'Connell JR, Weeks DE. The VITESSE algorithm for rapid exact multilocus linkage analysis via genotype set-recoding and fuzzy inheritance. *Nat Genet.* Dec 1995;11(4):402-408.
10. Abecasis GR, Cherny SS, Cookson WO, Cardon LR. Merlin--rapid analysis of dense genetic maps using sparse gene flow trees. *Nat Genet.* Jan 2002;30(1):97-101.
11. Zhou H, Pan W. Binomial mixture model-based association tests under genetic heterogeneity. *Ann Hum Genet.* Nov 2009;73(Pt 6):614-630.
12. Dempster AP, Laird NM, Rubin DB. Maximum likelihood from incomplete data via the EM algorithm. *J Roy Stat Soc B.* 1977;39(1):1-38.
13. Ott J. Linkage analysis and family classification under heterogeneity. *Ann Hum Genet.* 1983;47:311-320.
14. Ott J. *Analysis of Human Genetic Linkage.* Third ed. Baltimore, MD: The John Hopkins University Press; 1999.
15. Huang J, Vieland VJ. The null distribution of the heterogeneity lod score does depend on the assumed model for the trait. *Hum Hered.* 2001;52(4):217-222.
16. Purcell S, Neale B, Todd-Brown K, et al. PLINK: a tool set for whole-genome association and population-based linkage analyses. *Am J Hum Genet.* Sep 2007;81(3):559-575.
17. Abel L, Muller-Myhsok B. Maximum-likelihood expression of the transmission/disequilibrium test and power considerations. *Am J Hum Genet.* Aug 1998;63(2):664-667.
18. Gordon D, Heath SC, Liu X, Ott J. A transmission/disequilibrium test that allows for genotyping errors in the analysis of single-nucleotide polymorphism data. *Am J Hum Genet.* Aug 2001;69(2):371-380.
19. Tu IP, Whittemore AS. Power of association and linkage tests when the disease alleles are unobserved. *Am J Hum Genet.* Feb 1999;64(2):641-649.
